# Remote and low-cost intraocular pressure monitoring by deep learning of speckle patterns

**DOI:** 10.1117/1.JBO.29.3.037003

**Published:** 2024-03-29

**Authors:** Zeev Kalyuzhner, Sergey Agdarov, Yevgeny Beiderman, Aviya Bennet, Yafim Beiderman, Zeev Zalevsky

**Affiliations:** a Bar-Ilan University, Faculty of Engineering and the Nanotechnology Center, Ramat-Gan, Israel; b Holon Institute of Technology, Faculty of Electrical and Electronics Engineering, Holon, Israel

**Keywords:** intraocular pressure, glaucoma, photonics, machine learning, remote sensing, biomonitoring

## Abstract

**Significance:**

Glaucoma, a leading cause of global blindness, disproportionately affects low-income regions due to expensive diagnostic methods. Affordable intraocular pressure (IOP) measurement is crucial for early detection, especially in low- and middle-income countries.

**Aim:**

We developed a remote photonic IOP biomonitoring method by deep learning of the speckle patterns reflected from an eye sclera stimulated by a sound source. We aimed to achieve precise IOP measurements.

**Approach:**

IOP was artificially raised in 24 pig eyeballs, considered similar to human eyes, to apply our biomonitoring method. By deep learning of the speckle pattern videos, we analyzed the data for accurate IOP determination.

**Results:**

Our method demonstrated the possibility of high-precision IOP measurements. Deep learning effectively analyzed the speckle patterns, enabling accurate IOP determination, with the potential for global use.

**Conclusions:**

The novel, affordable, and accurate remote photonic IOP biomonitoring method for glaucoma diagnosis, tested on pig eyes, shows promising results. Leveraging deep learning and speckle pattern analysis, together with the development of a prototype for human eyes testing, could enhance diagnosis and management, particularly in resource-constrained settings worldwide.

## 1 Introduction

Glaucoma is a chronic and progressive eye disease leading to damage of the optic nerve and blindness. It comes in a variety of forms. Of these, open-angle glaucoma (OAG), normal tension glaucoma, angle-closure glaucoma, pigmentary glaucoma, and trauma-related glaucoma are the most prevalent.^1^^–^^6^ Ongoing research aims to gain a more comprehensive understanding of the distinction between normal tension glaucoma under normal intraocular pressure (IOP) and ocular hypertension without causing the illness. This research delves into various aspects, including diurnal tension variations, progression comparisons between untreated patients with normal-tension glaucoma and those with therapeutically reduced IOPs, and circadian IOP patterns in healthy subjects and glaucoma patients. These investigations are vital for advancing our knowledge of the complex dynamics of IOP and its role in glaucoma pathogenesis.^4^^–^^6^

IOP exhibits dynamic physiologic fluctuations with both regular circadian rhythms and random variations occurring over short and extended periods. These fluctuations are influenced by the subject’s muscular tone and physiologic state.^1^^,^^3^^–^^5^^,^^7^^–^^9^ Therefore, ensuring reliable IOP monitoring is of paramount importance in the clinical management of glaucoma. Despite the crucial role of IOP in guiding decisions related to glaucoma, contemporary glaucoma monitoring primarily relies on frequent IOP assessments during office hours. However, this approach often provides limited diagnostic adequacy, primarily due to the inherent fluctuating nature of IOP.^3^^,^^10^

Goldmann applanation tonometry (GAT) is the most frequently used ophthalmic tool for measuring IOP.^11^^–^^13^ Although GAT is precise, it is influenced by inner-individual variances, owing to differences in corneal thickness and stiffness.^13^ The method is intrusive and necessitates the administration of anesthetic eye drops, limiting IOP monitoring over time. Corneal biochemical characteristics impacting the accuracy of the applanation tonometry. The ocular response analyzer (ORA) allows for the IOP adjustment by considering the biomechanical parameters of the cornea.^12^^–^^15^ By directing an ultrasonic wave to the surface of an eye, researchers have been able to evaluate biological pulses, blood flow,^16^ and resonance modes of the eye cornea under a sound wave stimulation.^17^ Even though such procedures employ sound-driven technology to measure the physical properties of the eyes, no association with IOP has been found.^10^^,^^18^ An alternative method of IOP measurement is based on the air puff tonometer, which evaluates IOP based on the resistance of the eye to the air puff.^19^ However, for several reasons, the method is not suitable for obtaining full IOP profiles over long periods. First, air puff measurements can be affected by several external factors, such as the patient’s eye position and movements and eyelid position, leading to an increased variability in IOP measurements. Second, the air puff method may not be suitable for assessing diurnal IOP variation, which is important for glaucoma diagnosis and treatment. To obtain a full IOP profile, multiple measurements are required over an extended period, which can be time-consuming and inconvenient for both the patient and the healthcare provider. Also, repeated use of the air puff can cause dryness and irritation of the eyes, leading to discomfort and reduced accuracy of the readings.

The above constraint has triggered the need for devising novel ways for continuous IOP monitoring. Several reported examples include implantable telemetric pressure transducers,^20^^–^^23^ sensing contact lenses,^6^^,^^24^^–^^28^ implantable microfluidic devices,^29^ ocular telemetry sensors,^30^ and optical devices.^31^^–^^34^

One laser-based system^35^ demonstrated the capacity to remotely recognize speech signals,^35^ heart beats,^35^ blood pulse pressure,^36^^,^^37^ blood oxygen saturation,^38^ and sensations.^39^^,^^40^ One method^35^ measures the speckle in the far field using a defocused camera to extract several bio-medical data from their dynamic. In this process, the tilting motion is transformed into a linear movement of the speckle patterns, which may be readily retrieved using correlation-based operations:

$$T_{m}(x_{o},y_{o})=\iint \exp[i\phi (x,y)]\exp[\frac{\pi i}{\lambda Z_{1}}((x-x_{o}{)}^{2}+(y-y_{o}{)}^{2})]dx\;dy=A_{m}(x_{0},y_{0})\exp[i\psi (x_{0},y_{0})],$$ (1)

where (x,y) are the coordinates of the transversal plane, the axial axis is denoted by Z, λ is the optical wavelength, ϕ is the random phase created by surface roughness, and $Z_{1}$ is the distance between the object and plane captured by imaging system, with the intensity of the obtained speckle image being

$$I(x_{s},y_{s})={\left|\iint T_{m}(x_{o},y_{o})h(x_{o}-Mx_{s},y_{o}-My_{s})\right|}^{2},$$ (2)

where h is the spatial impulse response, M is the inverse of the magnification of the imaging system, and $\left(x_{s},y_{s}\right)$ is the sensor plane coordinate set.

A prior method involved remote IOP evaluation using the speckle pattern analyses of sound wave-stimulated fisheye records.^41^ The method evaluates IOP by analyzing the damping factor of the free sclera oscillations.^41^ However, the mistake of the IOP measurement in the range 15 to 25 mm Hg could reach 5 mm Hg.

We propose remote photonic IOP biomonitoring based on temporally encoded external sound wave stimulation, which does not require direct contact with the eye and is inexpensive to build. The suggested configuration includes projecting a laser beam onto the eye sclera stimulated by a sound wave, recording the secondary speckle patterns with a fast-imaging camera, and subsequent data processing using AI methods. To effectively reduce and manage background noise from the recorded signal, we developed a deep learning-driven IOP measurement model. This model enables in-depth analysis of the recorded signal, taking into consideration not only the free oscillations of the eye sclera but also the forced oscillations induced by periodic stimulation.

The method was successfully tested on 24 pig eyeball samples, given their similarity to the human eye.^42^^,^^43^ The tests were conducted by artificial variation of IOP. The method demonstrated a possibility for detecting changes in IOP. This approach does not necessitate preliminary calibration to verify the precision of the measurement devices and shows that the technique may be applied without extensive preparation or the specialist knowledge.

## 2 Materials and Methods

### 2.1 IOP Biomonitoring System Design

The pig eye has become a popular research model due to the ethical and financial constraints involved in employing eyes of other species.^42^^,^^43^ Pig eyes are very similar to human eyes, having holangiotic retinal vasculature, no tapetum, cone photoreceptors in the outer retina, and similar scleral thickness.^44^

The 24 tested pig eyeballs were acquired from a local distributor within less than 2 h postmortem, and the experiments were performed within 8 h following delivery. To preserve biological tissues by preventing further decay and degradation, the eyeballs were fixed with 4% paraformaldehyde in 0.1 M phosphate buffer saline (PBS, pH 7.4) for 4 h at 4°C, subsequent to which the retinas were removed and flat-mounted with the retinal ganglion cell layer uppermost. Then, the eyes were cover-slipped with PBS/glycerin (1:1). It is essential to note that, during the actual sampling and testing phases, we used clean eyeballs without any barriers or cover-slips. This approach allowed for maintaining the integrity of the eyeballs while ensuring no obstructions or barriers during data collection.

**Fig. 1:**
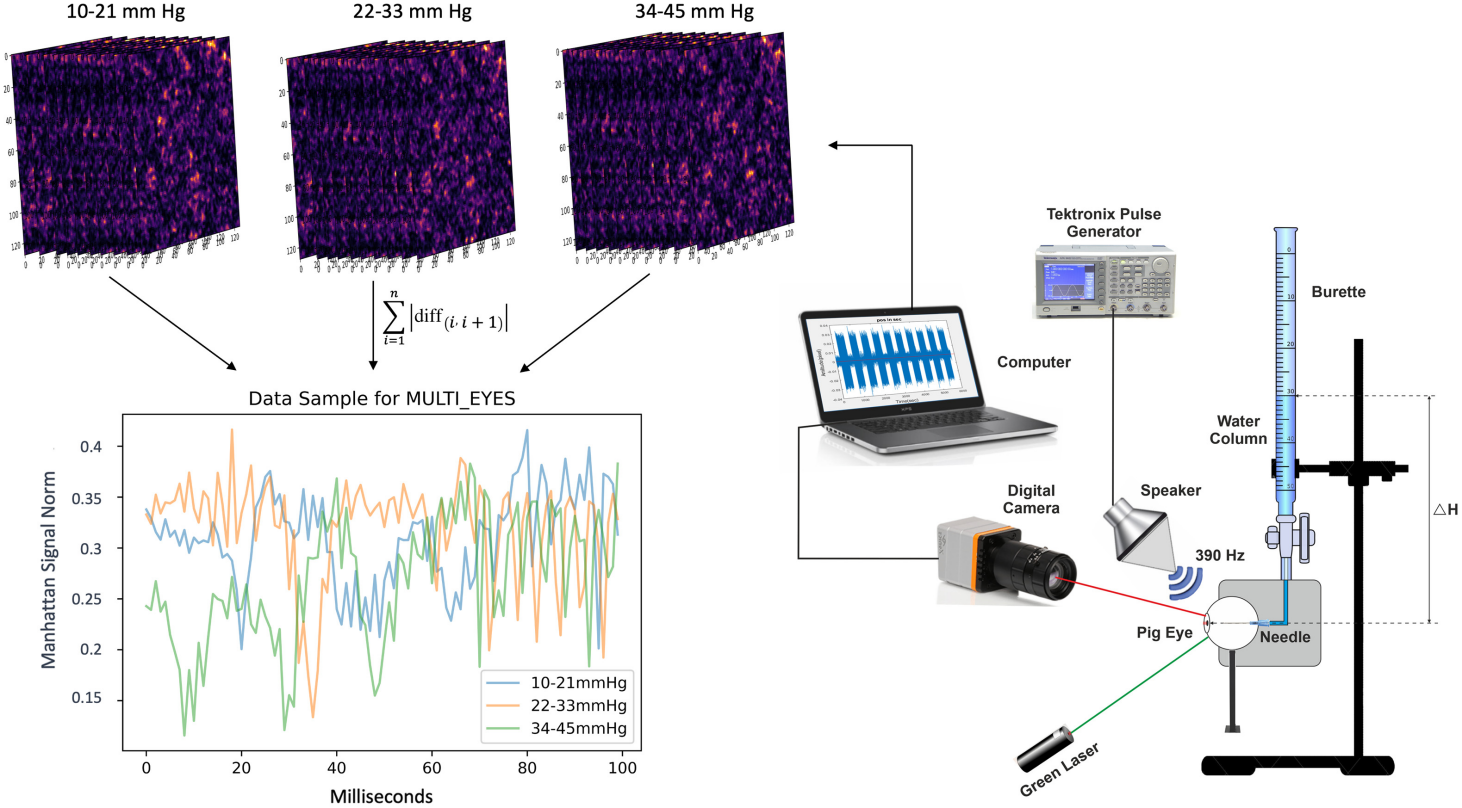
IOP biomonitoring system setup, simulation, and preprocessing.

The optically based monitoring device was positioned at a distance of 35 cm from the tested pig eyeball; see Fig. 1. Because speckle diffraction occurs over a wide angle,^35^ no constraints concerning the position of the fast-imaging camera exist.

We utilized a 532 nm continuous-wave (CW) green laser with a maximum power output of 300 mW (Model PPGL-2100F CW 300 mW max). The laser was carefully positioned directly opposite the illuminated eyeball, as shown in Fig. 1. The laser beam covered a 3 mm diameter area and was fixed at a selected location on the sclera adjacent to the pupil. It is noteworthy that the applied laser power of 750  μW falls within the safe range for human eyes, ensuring the safety of our experimental setup.^45^

For the recording of speckle patterns reflected from an eye sclera, we employed a Basler ace acA1300-200um digital camera configured to capture frames at a rate of 1000 frames per second (FPS). The camera was set with a spatial resolution of 64×64  pixels, with a focal length of 55 mm, and an F-number of 2.8, using a Basler C23-5026-2M-S f50mm lens. We maintained the camera’s focus on a far field, inducing defocusing of the sclera and the speckle pattern. The frame exposure used for speckle patterns recording was 200  μs. The defocusing technique caused the speckle pattern to move solely in the transversal plane.

To stimulate the sclera, we employed a loudspeaker (Pioneer, Ts-G1615R) set for the excitation frequency of 390 Hz@105 dB.^41^ The speaker was found to be highly responsive after a sweep of frequencies between 130 and 1000 Hz. An arbitrary waveform function generator (Tektronix, AFG3022B) controlled the speaker, as shown in Fig. 1. For each recording, the sound wave agitated a pig eye by 26 consecutive cycles, each cycle comprising one second of stimulation followed by 1 s of a break. The frame rate of the digital camera was more than twice the stimulation frequency to meet the Nyquist ratio requirements. Each frame of the camera output contained a secondary speckle pattern.

The eye pressure was actively controlled using a water filled transparent burette connected to a pig eye by an injection needle. The water column pressure was expressed in mm Hg with a 1 mm resolution. Each 1 mm Hg was considered equivalent to a 13.2 mm water column. The normal human eye pressure falls within the range of 10 to 21 mm Hg.^46^ Each eye was tested with a single needle penetration, and the IOP was adjusted at intervals of 1 mm Hg within the range of 10 to 21 mm Hg to ensure high accuracy within the normative pressure range. For pressures exceeding 21 mm Hg, testing was conducted with 2 mm Hg intervals. The maximum IOP value was defined at 45 mm Hg. Each tested eye was rejected after one day of complete testing under singular needle penetration.

### 2.2 Data Processing

The tests were conducted on separate days upon receipt of the samples, and each pig eyeball was tested in one continuous session. The dataset comprised ∼20 million frames captured from 24 pig eyes, each contributing ∼15 distinct videos with varying IOP ranges. Each video lasted 52 s (equivalent to 52,000 frames at 1000 FPS). Each video frame comprised a two-dimensional array with a spatial resolution of 64×64  pixels. Notably, for each test sample, corresponding to a specific eyeball and IOP range, we had at least one video with the same setup. Each pig eyeball video was given a unique identification containing the duration of the measurement and the IOP reference value.^40^

The videos from different recording days were initially combined to form an overall dataset. From this dataset, we meticulously created distinct subsets for training and testing purposes.^39^ During the subdivision, we ensured that frames from the same eyeball were exclusively assigned to either the training or test set, maintaining the separation to avoid any mixing or leakage of data between the two sets. By following this approach, we aimed to include data from all tested eyeballs in the analysis, while upholding the appropriate evaluation protocols, with a train-test split ratio of 80:20, ensuring a substantial portion of data for robust testing and evaluation.

IOP classification was divided into two testing components. The first component, the generic remote IOP classification, determined IOP in a discrete manner, requiring no calibration or prior knowledge about the tested eye. The main goal of this component was to identify abnormal IOP levels for further examination. The classification system subdivided the input signal into one of three possible classes with a range of 12 mm Hg. The first class was the normal range of 10 to 21 mm Hg. The second class, representing high IOP, encompassed the range of 22 to 33 mm Hg. The third class, representing extremely high IOP, covered the range of 34 to 45 mm Hg. A combined data sample of the three IOP ranges is shown in Fig. 1. It can be observed that the signal contains two visible components that affect the setup: a short period around 2 to 3 ms corresponding to the agitation frequency of 390 Hz and a prolong period around 75 Hz, corresponding to the frequency of 13 Hz, related to the frequency coming from the driver of the laser. The mentioned frequencies are dominating on the graphs related to the three ranges of IOP.

The second component determined the IOP of each tested eye with an accuracy level of 1 mm Hg, utilizing prior calibration, which involved establishing a calibration model using data from previous tests. The process involves training a model for each eye. To prove the feasibility of the method, we focused on the normal IOP range of 10 to 21 mm Hg. A set of IOP sensitivity techniques was defined so that each successive technique improved the accuracy over its predecessor. The first technique was a binary classification task that classified two IOP ranges: 10 to 15 mm Hg and 16 to 21 mm Hg, each having a 6 mm Hg range. The second technique was able to classify three different IOP ranges: 10 to 13, 14 to 17, and 18 to 21 mm Hg. The third technique was able to classify the exact measured IOP level with a deviation range of 1 mm. Each model was tested for each eyeball and compared with all tested eyeballs.

The generic and individual components of IOP monitoring allowed for the design of an IOP classification system that is accurate, permitting rapid identification of both abnormal and normal IOP.

To classify the IOP, each video, containing 52,000 frames, underwent pre-processing for frame correlation extraction (Fig. 1). For every two consecutive frames, the correlation was calculated using a full discrete two-dimensional linear cross-correlation^19^ with symmetrical boundary conditions, representing the shift between the two frames. To normalize this correlation signal, the Manhattan norm^19^ was applied using the following equation:

$$\text{norm}=\overset{n}{\underset{i=1}{\sum }}|{\mathrm{diff}}_{\left(i,i+1\right)}|,$$ (3)

where diff represents the correlation between two consecutive frames and “label” corresponds to the sample i. The Manhattan norm, as expressed in Eq. (3), sums the absolute differences between consecutive frame correlations. As part of the output, a one-dimensional (1D) array was created by pre-processing all frames of each recorded video.

A quantitative assessment and comparison of our proposed method used the metrics shown in Eqs. (5)–(8), where TP is the true positive, TN is the true negative, FP is the false positive, and FN is the false negative, which are calculated on a per-frame basis by the logical operators given in Eq. (4):

$$TP_{i}=(x_{i}==1)\&(y_{i}==1)\;TN_{i}=(x_{i}==0)\&(y_{i}==0)\;FP_{i}=(x_{i}==1)\&(y_{i}==0)\;FN_{i}=(x_{i}==0)\&(y_{i}==1),$$ (4)

$$\text{accuracy}=\frac{1}{n}\overset{n}{\underset{i=1}{\sum }}\frac{TP_{i}+TN_{i}}{TP_{i}+TN_{i}+FP_{i}+FN_{i}},$$ (5)

$$\text{precision}=\frac{1}{n}\overset{n}{\underset{i=1}{\sum }}\frac{TP_{i}}{TP_{i}+FP_{i}},$$ (6)

$$\text{recall}=\frac{1}{n}\overset{n}{\underset{i=1}{\sum }}\frac{TP_{i}}{TP_{i}+FN_{i}},$$ (7)

$$F_{1}=\frac{1}{n}\overset{n}{\underset{i=1}{\sum }}\frac{2TP_{i}}{2TP_{i}+FN_{i}+FP_{i}}.$$ (8)

The tuple $\left(x_{i},y_{i}\right)$ is the model prediction and the label for sample i.

It is essential to emphasize that all experiments were conducted in strict adherence to established guidelines and regulations. Our experimental setup remained completely safe for biological tissues.

### 2.3 Deep Learning for IOP Classification

**Fig. 2:**
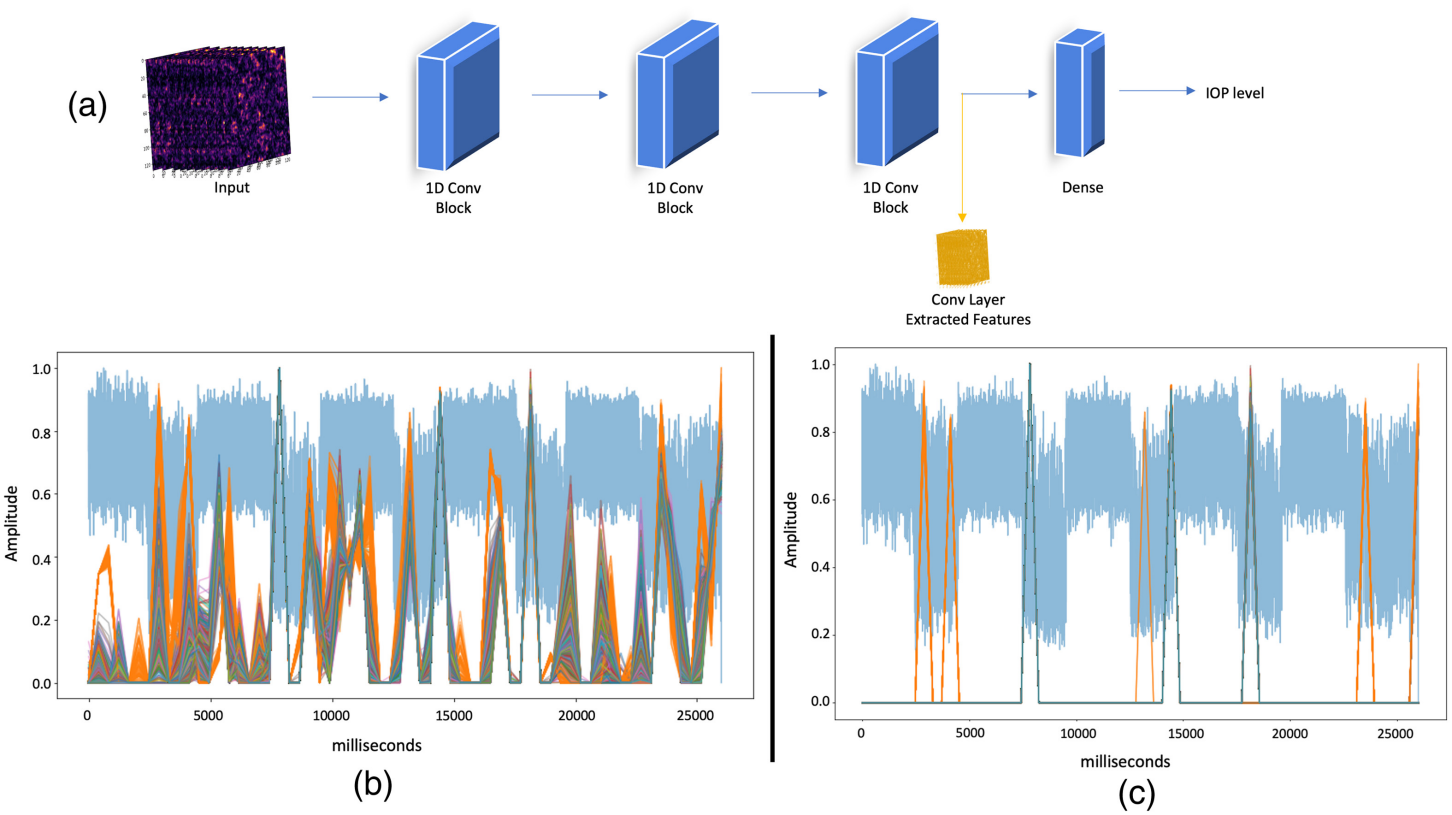
CNN model architecture and feature extraction sample. (a) CNN model architecture. The behavior of our model’s decision-making process could be clarified by extracting our trained convolutional layer filters and displaying them on a new input signal. (b) The input signal had a duration of 25,000 ms and was normalized to represent 26 consecutive cycles in the 0 to 1 range. Furthermore, our trained 64 convolutional layer filters constituted the CNN’s final convolutional layer, with the amplitude expressing the decision’s confidence level, with 1 being highest and 0 being lowest. Filters with amplitudes less than 0.8 were used after filtering layers. (c) When the eyeball started or stopped reacting to the external stimulation sound signal, the CNN classified the IOP level. Every increment in the IOP level induced alterations in ocular weight, volume, and geometry, thereby exerting a consequential influence on the configuration, orientation, and velocity of ocular motion, each of which was modulated by the auditory signal. From this physiological explanation, it is possible to conclude that the IOP level can be determined by remotely sensing nano-vibrations of an eye caused by an external sound stimulation.

The model’s input data for IOP classification are a 1D array, which is essentially a vector containing cross-correlation of the recorded consecutive video frames. These data were used to train a four-layer convolutional neural network (CNN) model for IOP classification, as shown in Fig. 2(a). The data were divided into training and testing sets, as explained in the previous section. The model was then applied separately to each of the three techniques for IOP classification.

The model output was specific to each technique, so we trained separate models for each technique. Each model was independently trained to output data according to its corresponding technique. The first three layers within the CNN model were a combination of 1D convolution,^47^ batch normalization,^48^ and rectified linear unit (ReLU),^49^ followed by a global average pooling operation.^50^^,^^51^ The last layer was a regular densely connected neural network (NN) layer with a Softmax activation function.^52^ The kernel size of each 1D convolutional layer was 3, with the corresponding 64 filters. The network output, representing the IOP classification resolution, depended on the specific IOP sensitivity technique. The loss function was categorical cross entropy:^53^

$$L_{\mathrm{CE}}=-\overset{n}{\underset{i=1}{\sum }}t_{i}\;\log(p_{i}),\;\text{for }n\text{ classes},$$ (9)

where $t_{i}$ is the truth label and $p_{i}$ is the Softmax probability for the i’th class.

For the training, the loss function was minimized by the Adam optimizer^54^ with β1=0.9, β2=0.999, and initial learning rate = 0.001. Using the reduced learning-rate on Plateau callback,^55^ we reduced the learning rate when the validation loss stopped improving. This deep learning procedure was implemented with a batch size of 32 for 500 epochs on a single 1080Ti graphics processing unit using a TensorFlow 2 package.

### 2.4 Comparison Between the Remote Photonic Validation and the Prior (*Q*) Factor Method

The prior method for IOP evaluation utilized the damping (Q) factor of the transitional free oscillations of the eye sclera surface following the termination of the stimulation by a temporally encoded sound wave.^19^ The obtained data were analyzed by the common statistical methods previously tested on artificial eyes and fisheyes.

To further validate the efficacy of the presented approach, we conducted experiments using a set of 24 pig eyes. Pig eyes were chosen as a suitable animal model for glaucoma research due to their similarities to human eyes under chronically increased IOP conditions. In addition, pig eyes are more accessible than nonhuman primate eyes.^19^

The presented approach does not require preliminary calibration for improved measurement precision. Using deep learning, we classified different IOP ranges accurately, without the knowledge of a specific IOP level.

Furthermore, although the prior method only analyzes the damping part of the free sclera oscillation, the presented method analyzes the entire signal, incorporating both the free and forced oscillation components. By considering the entire signal, we extract more comprehensive and robust data, leading to enhanced accuracy in the classification rate, using deep learning.

The presented novel method eliminates the need for preliminary calibration by incorporating the complete signal analysis. This technical improvement enhances the accuracy and reliability of the classification process, making the presented method a promising approach for noninvasive IOP monitoring.

## 3 Experimental Results

### 3.1 Results of the Generic IOP Classification

**Fig. 3:**
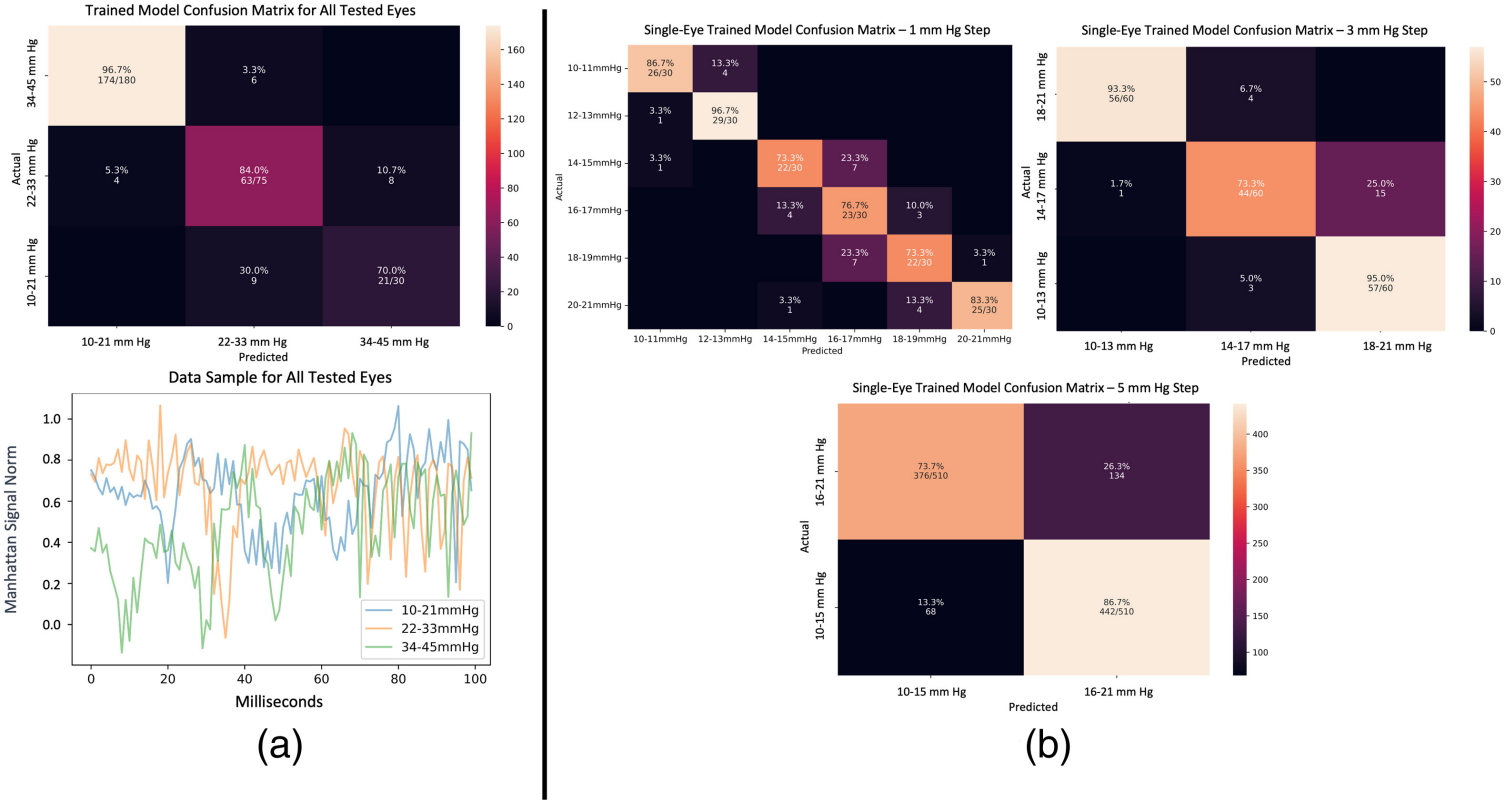
Confusion matrices and data sample plot of IOP trained models. (a) Confusion matrix of the generic model and a 100-ms pre-processed speckle pattern signal from all eyes. The average IOP range detection is 97% with a low error rate. High IOP ranges are identified at 84% (22 to 33 mm Hg) and 70% (34 to 45 mm Hg) with errors primarily occurring between these ranges. In addition, a data sample plot is provided to visually represent the input signal. (b) Confusion matrices of the three IOP classification tasks on a single eye. The presence of errors in close IOP ranges indicates successful learning. Specifically, in the 1 mm Hg IOP range variation, errors predominantly occur in the middle ranges and have relatively low values.

Generic remote IOP classification does not require any prior knowledge, and the model predicts the IOP accurately. Classification of the speckle patterns and their association with a specific IOP level was carried out using CNN. The results of the evaluation on an independent test set, to which the model was never exposed during training or validation, are given in Table 1, demonstrating that our model achieved an accuracy of 91%. In Fig. 3(a), we present a confusion matrix to elucidate the performance of our trained generic model. This matrix is based on a 100-ms data sample of pre-processed pig eye speckle pattern displacement, employed here as an illustrative example. It is imperative to underscore that our experimental protocol entailed the utilization of a complete 25,000-ms duration of the input signal, encompassing 26 consecutive data cycles. The depicted confusion matrix shows compelling insights into the IOP detection performance. Specifically, for a single test sample within the normal IOP range, we observed a remarkably high accuracy of 97%, accompanied by a notably low error rate. Moreover, the model demonstrates commendable success rates in identifying elevated IOP ranges, achieving accuracies of 84% for the 22 to 33 mm Hg range and 70% for the 34 to 45 mm Hg range, with the majority of errors confined to the range between 84% and 97%. Figure 3(a) also contains a graphical representation of these findings, with each IOP range visually differentiated by a distinct color. Table 1 reveals that, within the normal IOP range, the amplitude variations consistently exhibit smaller magnitudes in comparison with the two high IOP ranges. This amplitude represents the filter’s characteristics, reflecting the confidence level of the IOP classification decision. The observed variance in amplitudes across different IOP ranges indicates that our definition of the IOP classification problem is accurate and supports the feasibility of the proposed method. Instead of providing specific measurements of the amplitudes, our algorithm utilizes CNN to analyze and identify the differences in amplitudes corresponding to the IOP ranges. These variations in amplitudes are indicative of the physiological changes occurring in the eye as the IOP level varies. By leveraging the power of the CNN, our method effectively captures and utilizes this information to accurately classify IOP levels. This reinforces the soundness and validity of our approach in addressing the IOP classification problem.

**Table 1:** Generic and individual components of IOP monitoring model classification results.

Method		IOP range (mm Hg)	Precision (%)	Recall (%)	F1 (%)	Accuracy (%)
Generic photonic IOP classification (without calibration or prior knowledge)		10 to 21	98	97	97	**91**
		22 to 33	81	84	82	
		34 to 45	72	70	71	
Individual eye IOP classification	5 mm Hg IOP range	10 to 15	85	74	79	**80**
		16 to 21	77	87	81	
	3 mm Hg IOP range	10 to 13	87	82	84	**83**
		14 to 17	78	76	76	
		18 to 21	85	91	87	
	1 mm Hg IOP range	10 to 11	82	79	80	**70**
		12 to 13	64	69	65	
		14 to 15	66	58	60	
		16 to 17	63	55	55	
		18 to 19	69	76	70	
		20 to 21	86	78	79	

The generic method, which classifies pig eyeballs without calibration or prior knowledge, attains an accuracy of 91%, while maintaining a high recall of 97% and high precision of 98% in the normal IOP range classification task. The individual eye component is divided into three IOP sensitivity ranges: 5, 3, and 1 mm Hg. The 5 mm Hg range attains an accuracy of 80% while maintaining a high recall of 87% for the 16 to 21 mm Hg range and 85% precision for the 10 to 15 mm Hg range. The 3 mm Hg range attains an accuracy of 83% while maintaining a high precision of 87% for the 10 to 13 mm Hg range and 91% recall for the 18 to 21 mm Hg range. The 1 mm Hg range attains an accuracy of 70% while maintaining a high precision of 82% for the 10 to 11 mm Hg range and 86% precision for the 20 to 21 mm Hg range.

### 3.2 IOP Classification for a Particular Pig Eye

Utilizing calibration and prior knowledge of the specific eye, our classification technique uniquely identifies each eye, enabling a more accurate estimation of IOP with a higher precision compared with the generic IOP classification method. The measurement procedures involve classifying IOP sensitivity into three categories: 5, 3, and 1 mm Hg. Table 1 presents the average measurement metrics for all IOP ranges in the 24 tested eyes, including precision, recall, and accuracy.

For the 5 mm Hg measurement step, two classes were considered: 10 to 15 and 16 to 21 mm Hg, achieving an accuracy of ∼80% in identifying IOP for each class. The second IOP classification, with a 3 mm Hg step, resulted in an accuracy of 83%. Notably, Table 1 highlights that the highest measurement metric values are observed at the edges of the IOP ranges, such as 10 to 13 mm Hg with an F1 score of 84% and under 18 to 21 mm Hg with an F1 score of 87%. These findings align with the classification under the 1 mm Hg IOP step. Specifically, within the 10 to 11 mm Hg IOP range, the model achieved a precision of 82%, whereas for the 20 to 21 mm Hg IOP range, precision reached 86%.

In Fig. 3(b), we present the confusion matrices for the three specific IOP classification tasks. These matrices show the insights of the model’s performance, which reveal a distinct pattern. Notably, the model tends to make errors primarily within close proximity to IOP ranges rather than evenly distributed across all possible IOP values. This pattern signifies the model’s effective learning process. Moreover, when examining the confusion matrix for the 1 mm Hg IOP step variation, we observe that the highest error rate occurs within the mid-range of IOP values rather than at the extremes. Remarkably, these errors exhibit relatively low values, further characterizing the model’s behavior concerning fine-grained IOP variations.

### 3.3 Explanation of IOP Classification Model Results

The CNN models utilized in this study were optimized during the training process, leveraging the advantages of 1D convolutional layers. These layers, known for their weight sharing, sparsity of the connection capabilities, and parameter efficiency, were found to be beneficial for the IOP classification tasks. A key advantage of the convolutional layers is the ability to extract relevant features. Figure 2(a) demonstrates this by visualizing the trained convolutional layer filters applied to a new input signal, providing insights into the decision-making process of the model.

To provide further details, the input signal used in our experiments had a duration of 25,000 ms and was normalized within the range of (0, 1) to represent 26 consecutive cycles, as shown in Fig. 2(b), which also displays the 64 convolutional layer filters that constitute the final convolutional layer of the CNN. Each filter’s amplitude reflects the confidence level of the decision, with a value of 1 indicating the highest degree of confidence and 0 indicating the lowest.

Figure 2(c) demonstrates the classification behavior of the proposed CNN when the eyeball exhibits a response or ceases to respond to the sound of the external stimulation signal, characterized by a periodic vibratory profile. These responses are associated with different IOP levels. It is important to note that each IOP level corresponds to variations in the eye’s weight, volume, and geometry, which directly influence its shape, direction, and speed of movement, all affected by the agitating sound wave. From a physiological perspective, it is reasonable to conclude that, by remotely sensing the induced micro-vibrations of an eye, the IOP level can be determined accurately.

The amplitude variations in Fig. 2(b) reflect the model’s confidence level in its decision-making process, and Fig. 2(c) demonstrates the CNN’s classification behavior based on the eyeball’s responses to sound stimulation, associated with different IOP levels and their physiological influences on eye movements.

## 4 Discussion

We developed and demonstrated a low-cost, remote photonic IOP biomonitoring method that addresses the limitations of current IOP measurement techniques. The existing methods, such as GAT and ORA, often require expensive equipment, close proximity, and physical contact with a patient, which can be resource intensive.^19^ The proposed method utilizes the CNN classification of speckle patterns reflected from the eye sclera, illuminated by a laser beam, during temporally encoded external sound stimulation.

Our noncontact IOP biomonitoring tool offers several advantages over traditional methods. First, it is relatively inexpensive to manufacture and operate, with estimated hardware material costs under US$5000 for mass production. The device has the potential to be compact and mobile, making it convenient for various clinical settings. Importantly, the method demonstrates high accuracy even without preliminary calibration or prior knowledge about the tested eye. We conducted successful IOP biomonitoring of 24 pig eyes, which share similar characteristics to the human eye,^19^ further validating the accuracy and potential clinical utility of our approach.

Our photonic-based diagnostic tool is designed to enhance current high-performance clinical IOP measurement devices by effectively addressing major challenges related to cost, the use of anesthetic eye drops that impact the tested individual, and the need for specialized equipment and procedures. GAT, the commonly used method for IOP examination, is accurate, but could be influenced by individual variations in corneal thickness and rigidity. It also requires the use of anesthetic eye drops, limiting continuous IOP monitoring.^19^ By contrast, our photonic-based system offers an accurate and non-invasive diagnostic IOP measuring tool. By projecting an eye-safe laser beam onto the eye sclera and capturing scattered secondary speckle patterns using a fast-imaging camera, we provide a reliable alternative for IOP assessment.

To vary the IOP for experimentation, we inserted an injection needle into the pig eyeball, connecting it to a calibrated burette filled with water. The IOP was set at intervals of 1 mm Hg within the range of 10 to 21 mm Hg to ensure high accuracy in the normative pressure range. For pressures above 21 mm Hg, we conducted testing with 2 mm Hg intervals, with a maximum IOP value set at 45 mm Hg. This approach allowed for establishing a ground truth and ensures the effectiveness and reliability of the data collection process.

The results of our generic IOP model demonstrated an accuracy of over 90% with near-perfect detection of normative IOP levels. This system holds great potential for early detection of glaucoma, enabling proactive monitoring of individuals with high IOP through an accurate personal IOP measurement system.

In addressing potential challenges related to the missing data and variations in the data size between the training and test sets, we implemented preprocessing steps for model training. Although our dataset does not contain missing data, due to meticulous recording and control measures, the theoretical consideration of addressing the missing data points aimed to enhance the robustness of the CNN model and improve its generalizability across different data sizes. Part of this preprocessing involved utilizing the Manhattan norm on the frames to further refine the model’s ability to handle variations in the data size. It is worth noting that increasing the amount of training data generally leads to improved model performance. Collecting extensive data for each subject, particularly in clinical settings, can be resource intensive. Therefore, identifying the minimum data requirements for the reliable IOP classification is a crucial consideration, and our findings suggest that, even with a relatively modest dataset, accurate classification of high IOP can be achieved. The data size required to train a model for each subject depends on the number of single IOP values that we aim to classify. As a rule of thumb, the minimum data needed to identify if the subject is suffering from high IOP consist of at least two video samples, equivalent to 104 s of recording data (104,000 video frames), which helps prevent overfitting or underfitting.

It is important to note that our experimental setup involved pig eyeballs, which closely resemble human eyes, ensuring a reliable data collection process and accurate ground truth representation. These experiments aimed to simulate clinically significant glaucoma cases and demonstrate the feasibility of our method.^19^ The data collection took place in a controlled environment, with the laboratory darkened and silenced to minimize potential interference from background noises.^19^

Regarding the insertion of a needle into the eyeball, we maintained control over the direction of insertion, although we did not directly validate the precise location inside the eye. Although it is plausible for some needles to experience partial blockage due to the living tissue of the eyeball, we did not observe any significant blockage. This observation is supported by the slight drop in the water level of the calibrated burette, indicating successful filling of the eye with water.

Compared with previous methods, our presented approach, which includes a comprehensive signal analysis, showcases superior performance in accurately classifying IOP ranges. This improvement stems from the ability to assess not only the damping part but also the entire signal, which encompasses both free and forced oscillation components. These enriched data enable enhanced accuracy and reliability in the classification process, making our method a promising approach for noninvasive IOP monitoring. Furthermore, our approach eliminates the need for preliminary calibration, simplifying the measurement process and increasing its accessibility for clinical use.

Although the experimental setup has demonstrated promising results, the clinical prototype for *in vivo* noninvasive monitoring will undergo further enhancements to address challenges, such as fast eye saccades and pulsation. The system will be miniaturized and tested on human eyes, with the frequency generator replaced by a compact unit. All instruments will be securely mounted on a solid base fixed to a patient’s head to eliminate the effect of voluntary movements. In addition, the computer used for data processing and model training will be replaced by a compact, cost-effective, and dedicated computer designed for real-time use. We will also explore the integration of an infrared laser as part of our ongoing efforts to optimize and enhance the system’s performance.

The clinical utility of our approach lies in its potential for personalized IOP monitoring systems. The models presented in Table 1 were trained on the data from individual eyes and tested by the measurements from the same eyes, leveraging the specific characteristics and patterns of each eye for accurate IOP classification. This concept of personalized monitoring offers benefits for scenarios in which continuous monitoring and early detection of IOP changes are crucial, such as in glaucoma management.

## 5 Conclusion

Our study presented the development of a photonic IOP biomonitoring system that utilized CNN analysis of remotely recorded speckle patterns reflected from an eye sclera subjected to a periodic sound wave stimulation. This novel approach offers several key advantages, including its contact-free nature, low-cost implementation, and mobility, making it highly suitable for practical clinical applications.

Through rigorous testing on 24 pig eyeballs using our hardware and software platform, we achieved high accuracy in detecting elevated IOP levels. This successful demonstration of IOP detection holds promising clinical implications, particularly in the diagnosis of glaucoma, a condition commonly associated with high IOP.

One of the significant contributions of our research is the potential to address unmet clinical needs, especially in low- and middle-income countries. The development of the photonic-based technology offers the possibility of democratizing IOP diagnosis by providing accessible and affordable solutions. This has the potential to make a substantial impact on the global healthcare, ensuring that individuals in underserved regions can receive timely and accurate IOP assessments.

Building upon our research findings, future development efforts will focus on the creation of a human friendly, compact, head-mounted IOP sensing device. Such a device would enhance the practicality and ease of use in clinical settings, enabling efficient and non-invasive IOP measurements.

## Data Availability

The data generated to support the findings of this study are available from the corresponding author upon reasonable request. The code is available at https://github.com/zeevikal/iop-pigeye-speckle.
